# Combating the Infodemic: A Chinese Infodemic Dataset for Misinformation Identification

**DOI:** 10.3390/healthcare9091094

**Published:** 2021-08-24

**Authors:** Jia Luo, Rui Xue, Jinglu Hu, Didier El Baz

**Affiliations:** 1 College of Economics and Management, Beijing University of Technology, Beijing 100124, China; luo.jia.621125@gmail.com; 2 Graduate School of Information, Production and Systems, Waseda University, Kitakyushu 808-0135, Japan; jinglu@waseda.jp; 3 LAAS-CNRS, Université de Toulouse, CNRS, 31031 Toulouse, France; elbaz@laas.fr

**Keywords:** COVID-19, infodemic data, misinformation identification, deep learning

## Abstract

Misinformation posted on social media during COVID-19 is one main example of infodemic data. This phenomenon was prominent in China when COVID-19 happened at the beginning. While a lot of data can be collected from various social media platforms, publicly available infodemic detection data remains rare and is not easy to construct manually. Therefore, instead of developing techniques for infodemic detection, this paper aims at constructing a Chinese infodemic dataset, “infodemic 2019”, by collecting widely spread Chinese infodemic during the COVID-19 outbreak. Each record is labeled as true, false or questionable. After a four-time adjustment, the original imbalanced dataset is converted into a balanced dataset by exploring the properties of the collected records. The final labels achieve high intercoder reliability with healthcare workers’ annotations and the high-frequency words show a strong relationship between the proposed dataset and pandemic diseases. Finally, numerical experiments are carried out with RNN, CNN and fastText. All of them achieve reasonable performance and present baselines for future works.

## 1. Introduction

Infodemic is a portmanteau between information and pandemic. It refers to a substantial increase in misinformation associated with a specific pandemic and its growth can occur exponentially in a short period of time [1]. Governments and professional institutions try to provide up-to-date information and emotional support to reduce anxiety [2]. However, it is hard to cover every aspect. Once there is the absence of information, imagination and worst-case scenarios rush in. Social media has a significant impact in spreading fears and panic during the COVID-19 outbreaks [3]. People simply fail to think sufficiently about whether or not the content is accurate when deciding what to share [4]. Although social media has dramatically increased the degree of credibility of personal opinions and allowed them to spread more rapidly, each re-tweet or each article shared in a public chat increases the background noise [5]. Finally, misinformation around COVID-19 was unprecedentedly amplified.

Apart from the previous viral outbreaks, misinformation around the COVID-19 is considered as the first social media infodemic in the field of infodemiology [3] and this phenomenon was prominent in China at the beginning of the COVID-19 pandemic. Infodemic may lead to poor implementation of public health measures and weaken a countries’ ability to stop the pandemic. To cope with this problem, many social media service providers have paid much effort to collect and verify infodemic. However, manual identification has to face an issue of delay while social media make infodemic go faster and further in a short time. Thus, it is critical to develop techniques for infodemic detection and use them to track infodemic automatically.

Some deep learning approaches were developed to detect Chinese misinformation efficiently [6,7,8,9] via training misinformation data. However, the precision rate was as low as 28% when the deep learning method was used for infodemic detection [10]. It is easy to tell that the poor performance comes from general misinformation and infodemic mismatch while there is still a lack of open-source high-quality infodemic detection dataset which is even worse for Chinese. To solve the shortage of large expert annotation datasets in the field, this paper aims to present a Chinese infodemic dataset by collecting and arranging the widely spread Chinese infodemic during the COVID-19 outbreak. The main contributions of our work are summarized as follows:

1. A Chinese infodemic dataset is introduced. To the best of our knowledge, this is the first Chinese infodemic dataset for misinformation identification.
2. The original imbalanced dataset is converted into balanced by exploring the properties of the collected records.
3. The validation of the proposed dataset is verified by intercoder reliability and word frequency while experiments are carried with a baseline for future works.

The remaining sections of this paper are organized as follows. Section 2 introduces related works. Section 3 displays the process of the data collection. Section 4 provides the details of the dataset construction. Section 5 illustrates dataset validation. Afterward, numerical experiments and results analysis are presented in Section 6. Finally, Section 7 states conclusions and future works.

## 2. Related Works

Misinformation is usually related to rumors or fake news. In recent years, misinformation automatic detection has caught lots of attention. Castillo et al. [11] focused on the credibility of newsworthy information by using two classifiers where the first one decided if an information cascade corresponded to a newsworthy event and the second one decided if it was credible. Abbasi et al. [12] studied the situations in which they could not assess the credibility of the content or the credibility of the information source and proposed a CredRank algorithm to measure user credibility. Yu et al. [6] designed a convolutional approach for misinformation identification, which could flexibly extract key features from content credibility and microblog posts. Wang et al. [8] displayed an event adversarial neural network to derive event-invariant features and detect fake news on newly arrived events. Experiments of the above-mentioned works were conducted on datasets collected either from Twitter or/and Weibo.

There are some datasets utilized for general misinformation automatic detection and most of them are built based on data shared on Twitter. Kwon et al. [13] chose some rumor topics and some non-rumor topics that circulated on Twitter for a period of 60 days starting from one day prior to a key date. Afterward, four annotators were used to classify each topic manually according to four randomly chosen relevant tweets and a list of URLs. Boididou et al. [14] identified a set of online resources for important events that manually marked related images as fake or real and collected tweets by using keywords, hashtags and specific periods via Topsy API. Finally, the gathered tweets were filtered to keep those containing at least one image from the predefined fake or real seed sets. A tree-structured conversation formed of tweets replying to the originating rumorous tweet was used in [15] to build two subtasks. Each tweet in subtask A is annotated as support, deny, query or comment through crowdsourcing while each tweet in subtask B is annotated as true, false or unverified by journalist members who checked trustworthy sources manually. All of these datasets are built with the help of additional manual annotations which are strongly individual dependent and time-consuming.

To the best of our knowledge, datasets utilized for Chinese misinformation automatic detection are built using Sina Weibo, the primary microblog service in China. Different from Twitter, a community management center [16] is set up where common users are encouraged to report suspicious microblogs and a committee composed of reputable users is organized to verify the cases as false or real. As the easiest way, Ma et al. [17] collected 2313 known rumor data from the Sina community management center and 2351 non-rumor events by crawling the posts of general threads that were not reported as rumors. In [18], 2601 false rumors and 2536 normal messages were gathered by the same scheme where each was required to have at least 100 reposts. In addition to text content, Jin et al. [7] gathered texts with images and duplicated images were removed by a near-duplicated image detection algorithm based on locality-sensitive hashing. Because of the Sina community management center, it is easy to collect misinformation. However, it is still hard to judge the credibility of normal messages.

In a short period of time, many COVID-19 related datasets [19,20] were released while most of them are generic and lack annotations or labels. A few of them were collected for COVID-19 misinformation detection. As the traditional data source, Memon et al. [21] used Twitter and its API to gather 4573 tweets and manually annotated them into 17 categories. Zhou et al. [22] labeled 1364 pieces of news as reliable and 665 as unreliable based on the news sources listed in NewsGuard and Media Bias/Fact Check. [23] adopted a hybrid strategy where 204 fake news and 3565 true news were labeled according to the news sources while 28 fake claims and 454 true claims were annotated by experts. Moreover, Li et al. [24] built up a multilingual dataset consisting of 3981 pieces of fake news content and 7192 trustworthy information from six different languages. However, none of these utilized Chinese ones for research to the best of our knowledge, and there is no Chinese dataset for combating COVID-19 misinformation.

The above-mentioned datasets were collected from real-world social media platforms and were widely used for general misinformation automatic detection. However, the publicly available infodemic detection dataset is still very rare and not easy to be constructed manually. Therefore, instead of developing deep learning methods, this paper aims to construct a Chinese infodemic dataset, “infodemic 2019”, by collecting and arranging widely spread Chinese infodemic during the COVID-19 outbreak.

## 3. Data Collection

Datasets used for the Chinese misinformation automatic detection are generally built using Sina Weibo. Thus, we crawled data from Sina Weibo microblogs. In order to ensure a quality dataset, only microblogs manually verified by the Sina community management center are taken into consideration. Moreover, we focus on the period from 21 January to 10 April 2020 as Wuhan, the first identified COVID-19 outbreak city, was in lockdown from 23 January to 8 April 2020. Since these microblogs are reported by common users, some of them were duplicates and were therefore removed. Furthermore, microblogs that are not related to the COVID-19 are eliminated as well.

WeChat has become a primary news source for Chinese Internet users and provides many mini-app programs that function as “apps within an app” keeping users inside WeChat even as they perform tasks they might otherwise do elsewhere. “Jiaozhen”, which means “to take something seriously” in Chinese, is a mini-app launched by WeChat for checking widely spreading rumors. Unlike Sina Weibo where misinformation is only tracked passively by users’ reposts within the platform, the WeChat mini-app “Jiaozhen” debunks misinformation actively without platform limitation. Moreover, the verification results are more authoritative as it is operated by in-house fact-checkers who work with professionals such as medical doctors and professors along with other organizations such as the local police and news media. Each of the collected popular ambiguous information is labeled either as true, false or questionable according to by-lines with attached supporting materials. The criterion and representative examples for each category are listed in Table 1 where the different criteria which were chosen within one category may be ambivalent. In addition, “Jiaozhen” has provided an independent collection for fighting the COVID-19 related misinformation after the outbreak. Therefore, we trawled all records gathered by this special collection until 31 March 2020, apart from those that are duplicated with microblogs gathered from the Sina community management center.

Finally, records gathered from the Sina community management center are categorized as false rumors and are labeled as 1 while records coming from the WeChat mini-app “Jiaozhen” are labeled as 0, 1, 2, respectively, in accordance with questionable, false, true. The raw dataset contains 797 records where 128 are labeled as 0, 600 as 1, 69 as 2. More details are listed in Table 2.

## 4. Dataset Construction

**Figure 1:**
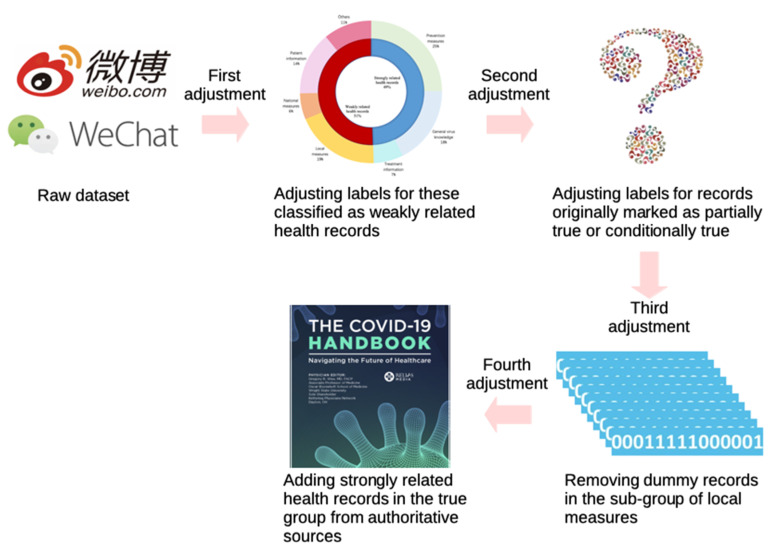
Process of four times label adjustment.

To avoid a “dummy” classifier that mistakenly classifies all records as false information and achieves high accuracy in classification (above 70%), a qualified infodemic dataset is supposed to contain roughly an equal number of records with each label. Therefore, we try to convert the raw dataset into balanced by four times adjustment. The whole process is depicted in Figure 1.

**Figure 2:**
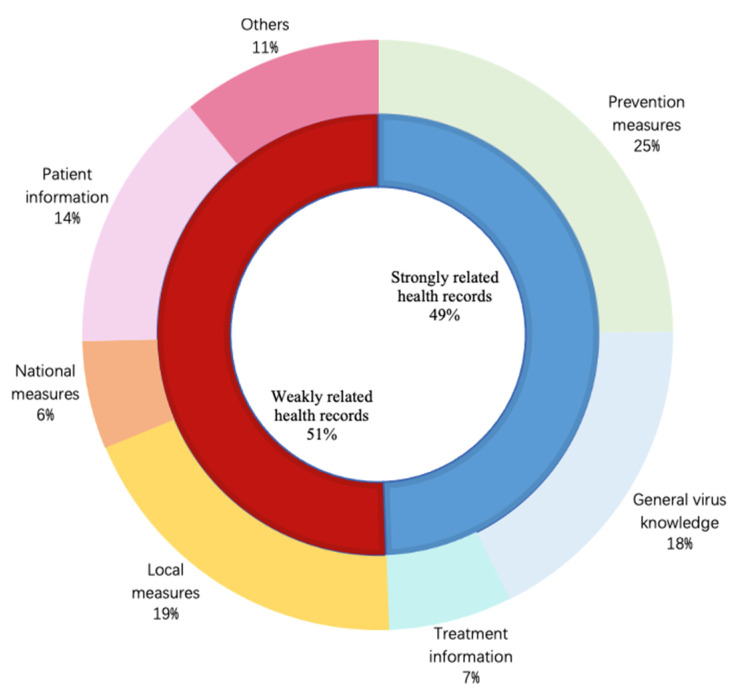
Proportion of different content types in the infodemic 2019 raw dataset.

As infodemiology focuses on scanning the Internet for user-contributed health-related content [25], we divide all instances into two types depending on the content: strongly related health records and weakly related health records. These are classified into three main stages of medical science: prevention, diagnosis and treatment, which are considered as strongly related health records while the rest are categorized as weakly related health records. In accordance with those three stages, the strongly related health records are further subdivided as prevention measures, general virus knowledge and treatment information [26]. After carefully analyzing the details of weakly related health records, we split this data into four sub-types as local measures, national measures, patient information and others according to involved action doers. The numerical proportions of the above-mentioned types and subtypes are displayed in Figure 2. Obviously, it is hard to verify the reliability of weakly related health records without checking who has completed them. As a result, we revise the labels of instances in this group as 0 even if their original labels are 1 or 2. After the first manual adjustment, the resulting dataset consists of 512 records with label 0, 249 with label 1 and 36 with label 2.

Concerning the criterion for the questionable category, records originally labeled as 0 are classified into four sub-categories as controversial, inconclusive, conditionally true and partially true. Common knowledge is mostly concerned with facts that are true or false under normal circumstances rather than with exceptions or very improbable cases. Thus, we revise the labels of strongly related health records marked as partially true from 0 to 1 or 2 by adding the conditions which make the underlying statement true or false. On the other hand, labels of strongly related health records marked as partially true are updated as 1, since a partial truth is usually considered as a deceptive statement. After the second manual adjustment, there are 478 records with label 0, 281 with label 1 and 38 with label 2 in the dataset.

Furthermore, we have found that there are some “dummy” records in the sub-type of local measures, where the content is similar while only the location information related to those records are changed: such as lockdown for different cities, school re-open time for different areas and so on. We decide to keep three records for the ones that have highly similar content. Consequently, the total amount of records labeled as 0 is decreased from 478 to 435 after the third manual adjustment. In addition, the number of records labeled as 1 is too few. Therefore, we have collected and edited 301 strongly related health records from the last versions of widely suggested official handbooks or authoritative webpages which focus on the COVID-19 prevention and treatment [27,28,29]. After four times manual adjustments, the infodemic 2019 dataset finally contains 1055 records where respectively 435 are labeled as 0, 281 as 1, 339 as 2. All records are saved in a CSV file with the following fields: ID, content, final label and original label (if it exists).

## 5. Dataset Validation

**Figure 3:**
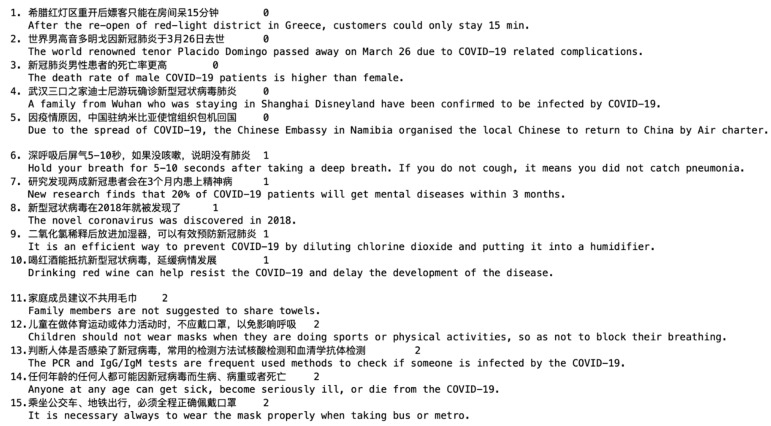
Representative instances of the infodemic 2019 dataset and the corresponding translation in English.

Fifteen representative instances with their final labels and the corresponding translation in English are shown in Figure 3. We find that records labeled as 1 or 2 are instances that can be identified as true or false by healthcare workers. However, it is hard for healthcare workers to estimate the confidence level of records labeled as 0. In order to verify this observation, we asked three healthcare workers as annotators to manually classify all records into three groups by their independent opinions without checking any reference. The manually annotated label of each record is decided by the majority agreement where at least two annotators have classified it into the same group.

There is high intercoder reliability [30] between labels after four times adjustment and labels annotated by healthcare workers. The details are shown in Table 3. Finally, instances labeled as 0 only contain records that require further checking with who has completed them and records that do not have a certain conclusion at that time. As a result, it is hard for health workers to judge them as true or false independently. Additional auxiliary features such as social engagements or user profiles are encouraged to be integrated as multimodal features in predicting their credibility. The proposed dataset is designed particularly for content-based methods with the goal of building an efficient classifier that could achieve the same performance as healthcare workers to decrease the total workload of manual identification.

**Figure 4:**
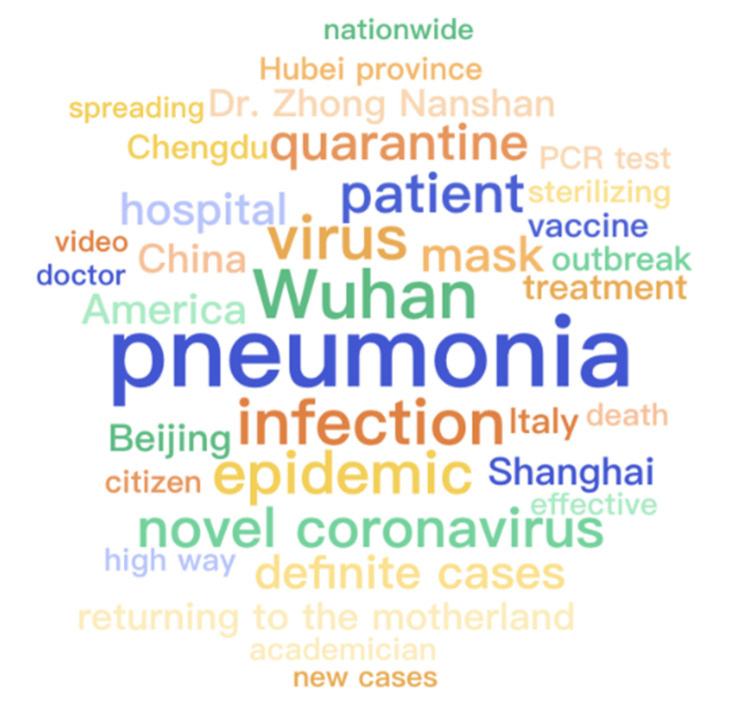
Word cloud of records labeled as questionable in the infodemic 2019 dataset.

**Figure 5:**
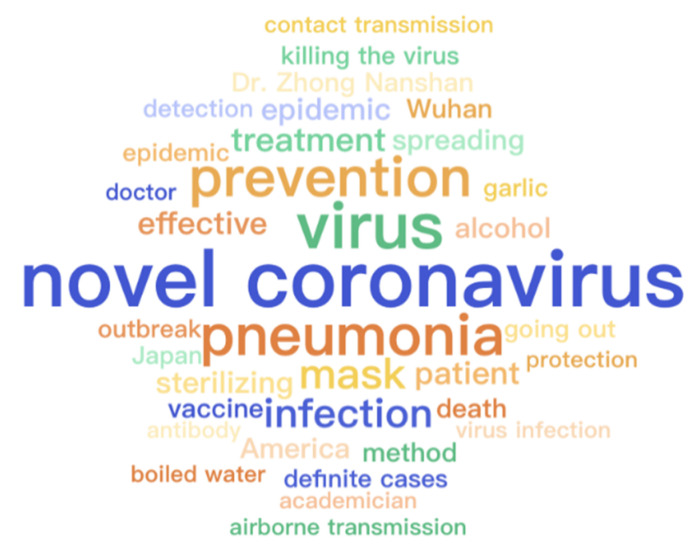
Word cloud of records labeled as false in the infodemic 2019 dataset.

**Figure 6:**
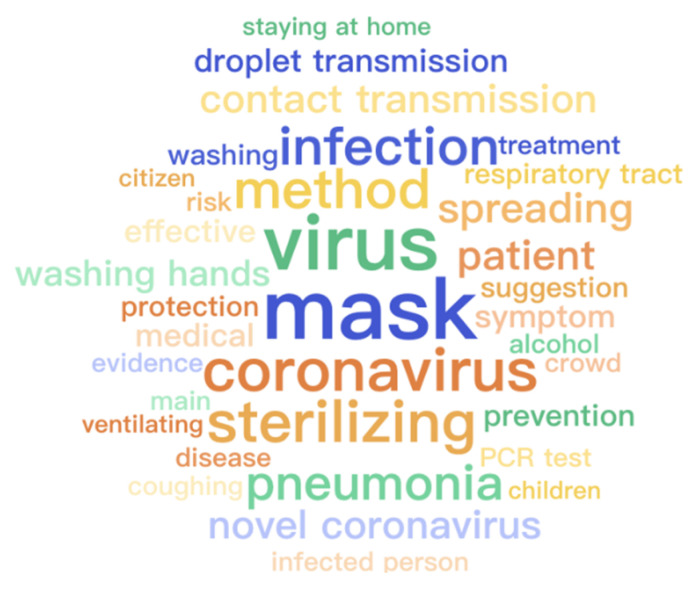
Word cloud of records labeled as true in the infodemic 2019 dataset.

**Figure 7:**
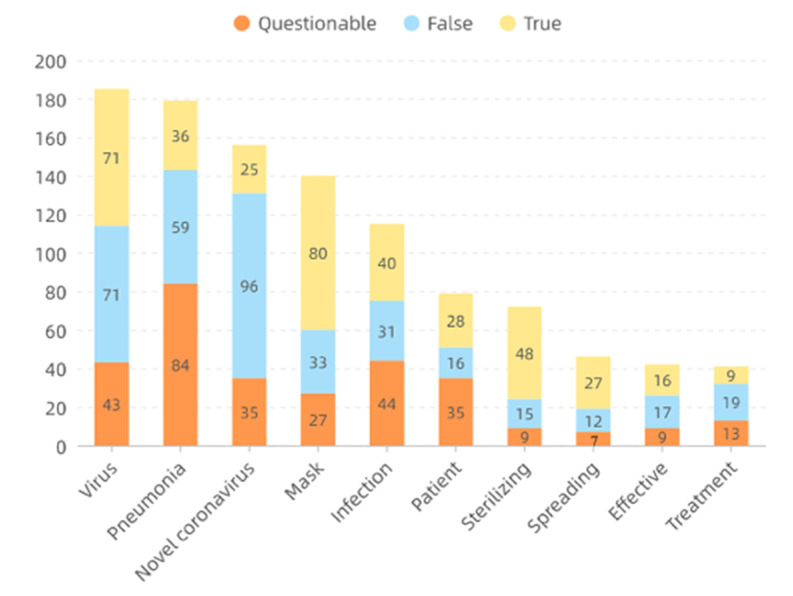
Frequently used words that appeared across three groups and their word frequency in each category.

The thirty-five most frequent words generated by [31] for each category are displayed in Figure 4, Figure 5 and Figure 6, respectively. All words are translated into English that are illustrated with font size scaled to their frequencies. Because of the first adjustment, words about specific locations are mentioned more frequently inside the questionable group while the other two groups contain more about medical-related words. As there is a topic difference among the three categories, some topic model methods can be applied to learn latent stance from topics and improve classification accuracy. However, it is not suggested to directly apply the topic difference to a single piece of text [32]. Therefore, it is strongly encouraged to develop more advanced methods and learn a better representation of these contents, particularly for false and true groups. Moreover, ten frequent words appeared across three groups and their word frequency in each category are detailed in Figure 7. It is easy to find that all of them are strongly related to the pandemic which is the main difference between infodemic and general misinformation.

## 6. Baseline Experiments

Three deep learning methods are used to conduct the baseline experiments: recurrent neural networks (RNN), convolutional neural networks (CNN) and fastText. The long short-term memory (LSTM) is a frequently used RNN architecture that can not only process single data points, but also entire sequences of data [7]. Therefore, it is well-suited for processing written nature languages. We set the size of the hidden units in LSTM as 64 and the number of the hidden layers as 4. Moreover, the bidirectional structure is used to enable the networks to have both backward and forward information at every time step. CNN is originally designed for image analysis with a shared-weights architecture and translation invariance characteristics. However, it has recently been shown to achieve impressive results in sequent data analysis [33]. The designed CNN consists of one convolutional layer and one max-pooling layer where the size of the filter is set as 2, 3, 4 and the number for each filter is kept as 64. FastText is a library for the learning of word embeddings and text classification [34] where each word is represented as a bag of character n-grams. In order to increase the quality of the solution, the concatenation of the usual unigram average with bigram and trigram vector averages [35] is utilized. Moreover, the number of hidden units in the hidden layer is fixed as 64.

We implement the above-mentioned three models by using PyTorch, an open-source machine learning library. All of them are trained by employing the derivative of the loss through backpropagation with respect to all of the parameters. The Adam algorithm is used for parameter updates. We empirically set the dropout rate as 0.5, the learning rate as 0.001, the batch size as 64 and the length of embedding layers as 50. Moreover, the Chinese character embeddings pre-trained on the open-source Sougou News dataset are used where the vocabulary size is 4762 and the embedding size is 300. In the following experiments, each neural network model is trained for 100 epochs with early stopping to report the results. We randomly choose 10% of the proposed infodemic dataset for model tuning and the rest 90% are randomly assigned in a 3:1 ratio for training and test. Similar to [6,7,8,9], accuracy, precision, recall and F-measure are adopted as the evaluation metrics to measure the performance of infodemic identification.

Classification results are presented in Table 4. The accuracy of RNN, CNN and fastText are around 70% which indicates that basic deep learning models can learn discriminative features on the proposed infodemic dataset effectively. In details, they achieve an F1-score (precision, recall) from 81.55% (82.08%, 79.25%) to 82.52% (85.00%, 82.08%) for questionable records, from 51.16% (50.77%, 51.56%) to 66.13% (68.33%, 67.19%) for false records and from 69.06% (66.67%, 71.64%) to 80.00% (79.41%, 80.60%) for true records. All three baselines perform well in identifying questionable records while fastText obtains the best result in identifying false records and CNN outperforms the other two models in identifying true records. Concerning the overall performance, CNN is the best. It outperforms RNN not only generally but also in all evaluation metrics for each category.

**Figure 8:**
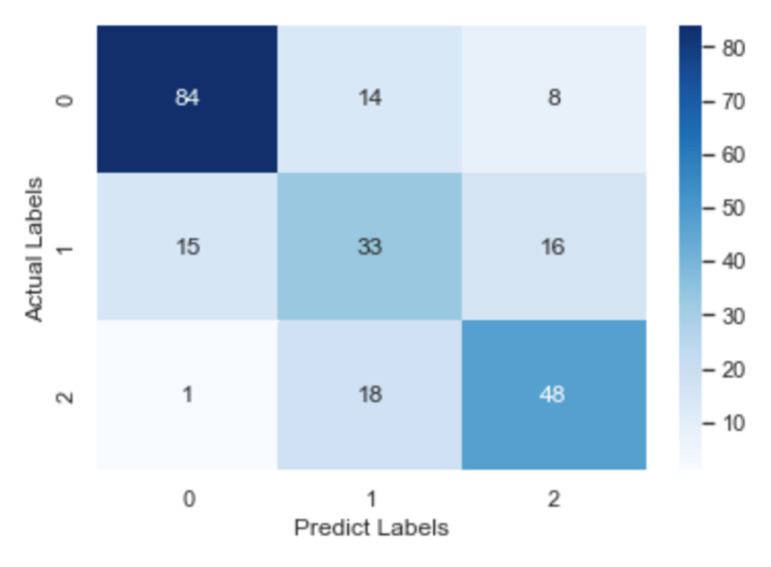
Confusion matrix of the RNN model.

**Figure 9:**
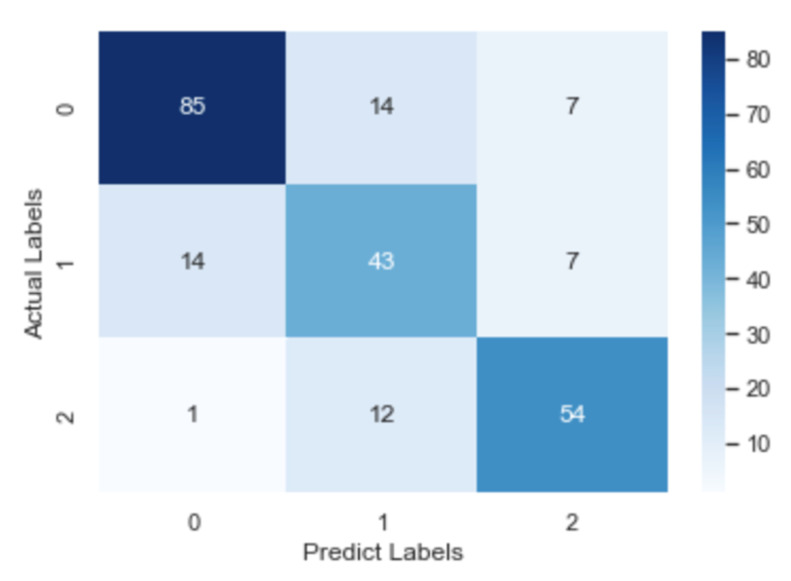
Confusion matrix of the CNN model.

**Figure 10:**
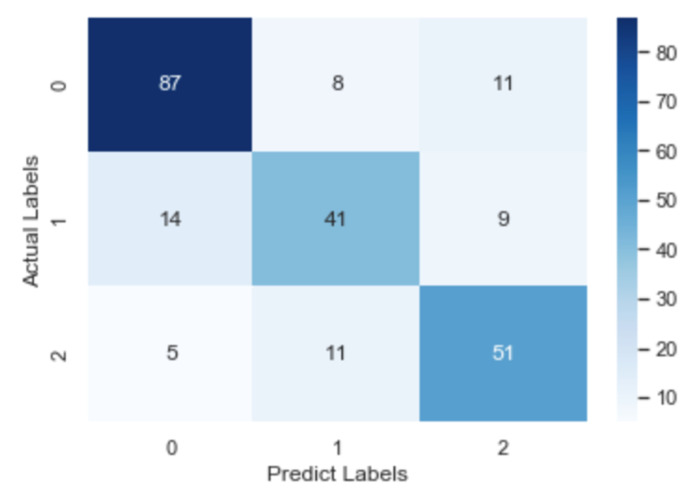
Confusion matrix of the fastText model.

To check further the details about misclassification, the confusion matrix of the CNN model, the RNN model and the fastText model is illustrated in Figure 8, Figure 9 and Figure 10, respectively. After an overall analysis of three figures, we can find that misclassification happens more frequently for false and true records. Additionally, false records are more frequently misclassified as questionable records while true records are more frequently misclassified as false records.

## 7. Conclusions and Future Works

In this paper, a Chinese infodemic dataset, infodemic 2019, was introduced. It contained 1055 records where respectively 409 were labeled as questionable, 276 as false, 335 as true. In order to fit the real-world applications, the original imbalanced dataset was converted into balanced by exploring the properties of the collected records. Labels of some instances were revised manually while “dummy” instances were eliminated and additional instances were added. The final labels achieved high intercoder reliability with healthcare workers’ annotation and the high-frequency words showed a strong relationship between the proposed dataset and pandemic diseases. Finally, numerical experiments were carried with RNN, CNN and fastText. All of them obtained an accuracy around 70% which indicated that basic deep learning models could learn discriminative features on the proposed infodemic dataset effectively.

This paper is just the start of infodemic automatic detection and three main areas that deserve further study are identified. The first issue is to implement other deep learning models to achieve higher accuracy than the baseline records. The second line of interest is to improve the overall performance by transfer learning along with cooperation between the proposed dataset and general misinformation identification datasets. Due to massive unlabelled data during the COVID-19 outbreak, we would like also to consider semi-supervised learning and unsupervised learning in future works.

## Figures and Tables

**Table 1:** Criterion and representative examples for records labeled as true, false or questionable in the WeChat mini-app “Jiaozhen”.

	Criterion	Representative Examples
Records labeled as questionable	Controversial	Remdesivir is efficient for the treatment of COVID-19.
	Inconclusive	Same as the flu, COVID-19 will outbreak seasonally.
	Conditionally true	The N95 mask needs to be changed every 4 h.
	Partially true	A virus can stick to hairs. Thus, it is necessary to wash hairs after arriving home.
Records labeled as false	False general knowledge	Perfume can be used to prevent COVID-19.
	False scientific knowledge	Drinking red wine can help resist COVID-19 and delay the development of the disease.
	Fake news	Wuhan’s coronavirus hospital will be relocated.
	Rumor	After the re-open of red-light district in Greece, customers could only stay 15 min.
Records labeled as true	General assertion	Family members are not suggested to share towels.
	True news	NBA announced the suspension of the 2019–2020 season.

**Table 2:** Composition of the infodemic 2019 dataset.

	N° of Records Labelled as Questionable	N° of Records Labelled as False	N° of Records Labelled as True
Raw dataset	128	600	69
Dataset after the first adjustment	512	249	36
Dataset after the second adjustment	478	281	38
Dataset after the third adjustment	435	281	38
Dataset after the fourth adjustment	435	281	339

**Table 3:** Intercoder reliability between labels after four times adjustment and labels annotated by healthcare workers.

	Questionable	False	True
Labels after four times adjustment	435	281	339
Labels agreed by healthcare workers	394	259	330
Intercoder reliability	0.9057	0.9217	0.9734

**Table 4:** Baseline experiment results in misinformation identification using the infodemic 2019 dataset.

Method	Metric	Questionable	False	True
RNN	Precision	0.8400	0.5077	0.6667
	Recall	0.7925	0.5156	0.7164
	F1-score	0.8155	0.5116	0.6906
	Accuracy	0.6962		
CNN	Precision	0.8500	0.6232	0.7941
	Recall	0.8019	0.6719	0.8060
	F1-score	0.8252	0.6466	0.8000
	Accuracy	0.7679		
fastText	Precision	0.8208	0.6833	0.7183
	Recall	0.8208	0.6406	0.7612
	F1-score	0.8208	0.6613	0.7391
	Accuracy	0.7553		

## Data Availability

The proposed dataset is available at https://www.dropbox.com/sh/praltzebemotd2r/AABmc1IxaKG_uZnEUN5beJFwa?dl=0 (accessed on 25 April 2021).
